# An Atypical Presentation of Median Arcuate Ligament Syndrome

**DOI:** 10.7759/cureus.7289

**Published:** 2020-03-16

**Authors:** Muhammad Shabbir Rawala, Amna Saleem Ahmed, Syed Rizvi

**Affiliations:** 1 Internal Medicine, Charleston Area Medical Center, Charleston, USA; 2 Internal Medicine, Jinnah Medical and Dental College, Karachi, PAK; 3 Cardiology, Rapides Regional Medical Center, Alexandria, USA

**Keywords:** celiac artery stenosis, median arcuate ligament, abdominal pain

## Abstract

Median arcuate ligament syndrome (MALS) is a rare condition where the celiac artery is compressed by the ligament, uniting the diaphragmatic crura of the aortic hiatus. Patients mostly present with abdominal symptoms. We present a case of a 51-year-old male who presented with abdominal pain. The patient was evaluated with a computed tomography (CT) scan of the abdomen and found to have celiac artery stenosis secondary to the median arcuate ligament (MAL). The patient was assessed by surgery and interventional radiology, but no intervention was offered. He was started on anticoagulation with spontaneous resolution of abdominal pain.

## Introduction

Median arcuate ligament (MAL) is a musculo-fibrous structure uniting the medial borders of the diaphragmatic crura on either side of the aortic hiatus [1-5]. The ligament passes above the origin of the celiac artery and is a direct continuation of the posterior diaphragm that wraps over the aorta [1]. In 10% to 24% of the population, an unusually low-lying MAL passes anterior to the celiac artery causing some degree of stenosis triggering abdominal symptoms [1,2,6]. Celiac trunk is most prone to constriction by MAL when the distance between the celiac trunk root and the diaphragmatic crura is short, i.e, an abnormally low-lying diaphragmatic MAL [7,8].

Median arcuate ligament syndrome (MALS), also known as coeliac axis compression syndrome or Dunbar syndrome, is a rare disorder resulting from the external compression of the celiac trunk by the MAL [1,4]. MALS is characterized by the triad of postprandial abdominal pain, weight loss, and often an abdominal bruit produced due to celiac artery compression by MAL [6].

Patients are usually 30 to 50 years old, thin females, who have had several workups for diagnosing the source of abdominal pain [1,4]. Most patients have incidental findings diagnosed on computed tomography (CT) scan and require no treatment [7]. Kuruvilla et al. mention the use of a mesenteric ultrasound during deep expiration as the modality makes use of the increased blood flow velocity developed in areas compressed due to celiac artery stenosis/constriction [6].

We present a case of a 51-year-old male who had presented to the emergency department with complaints of intermittent substernal chest pain and abdominal pain.

## Case presentation

The patient is a 51-year-old male who had presented to the emergency department with complaints of intermittent substernal chest pain that had been radiating to the left neck and arm for five days. He also had complaints of intermittent abdominal pain. He had co-morbid conditions of mesenteric artery thrombosis, left testicular cancer, and chronic obstructive pulmonary disease. The patient was evaluated at another facility a week ago and diagnosed with mesenteric artery thrombus. A repeat computed tomography angiography (CTA) was performed that identified mesenteric artery thrombosis and celiac artery stenosis.

On examination, the patient's cardiac examination was unremarkable; however, there was some tenderness in the abdomen without any rebound rigidity, guarding, or frank peritoneal signs. The patient was admitted and evaluated by the cardiology service; his troponins had been negative, an echocardiogram showed normal left ventricle, right ventricle, and diastolic function, he was started initially on aspirin, statin and heparin intravenous (IV) drip for mesenteric artery thrombosis. Invasive angiography (IA) was performed that revealed severe non-obstructive lesions in all three major epicardial arteries, and severe 90% lesion at the ostium of patent ductus arteriosus (PDA) vessel. The patient had opted for medical management, therefore optimized with aspirin, statin, ace-inhibitor, and isosorbide mononitrate.

**Figure 1:**
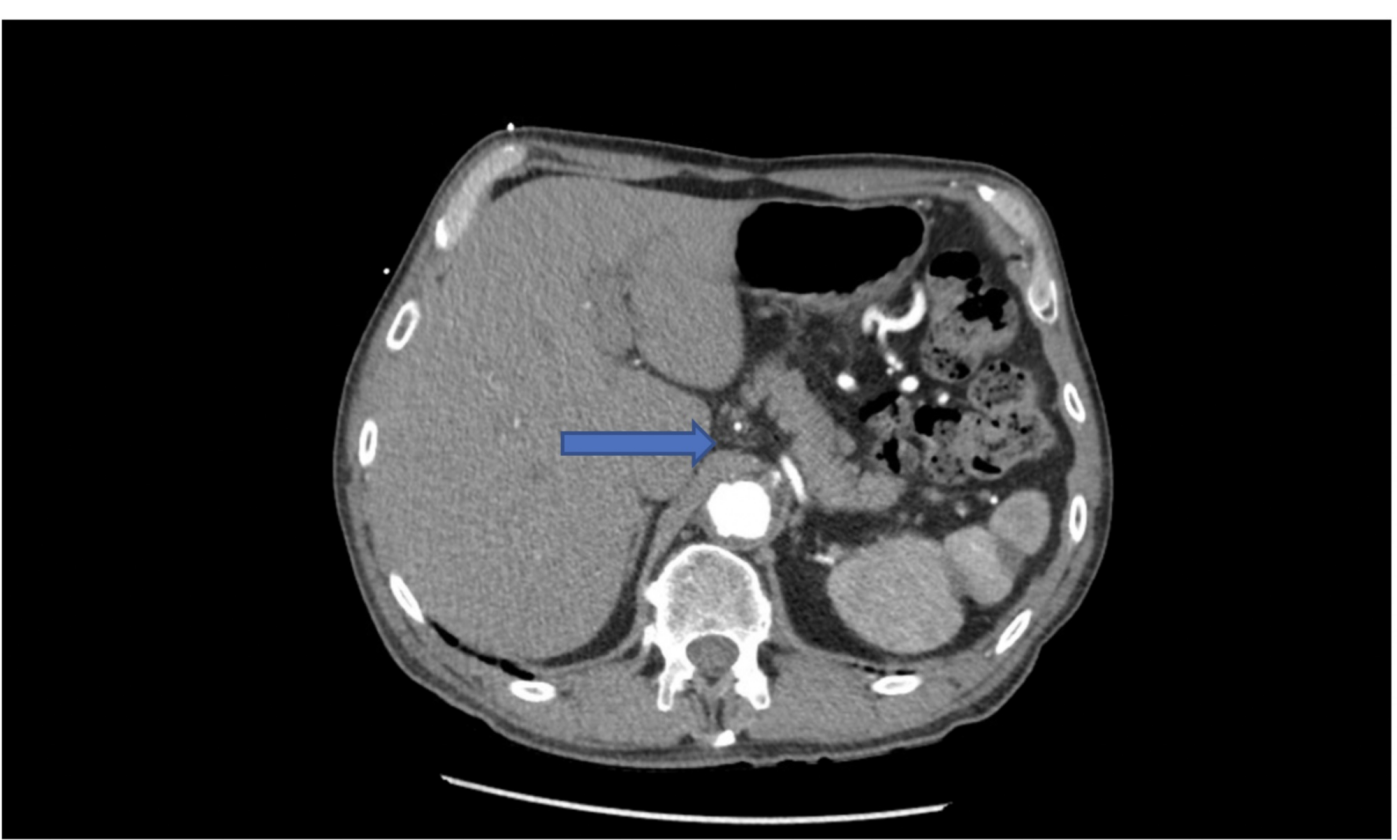
Median Arcuate Ligament (arrow)

**Figure 2:**
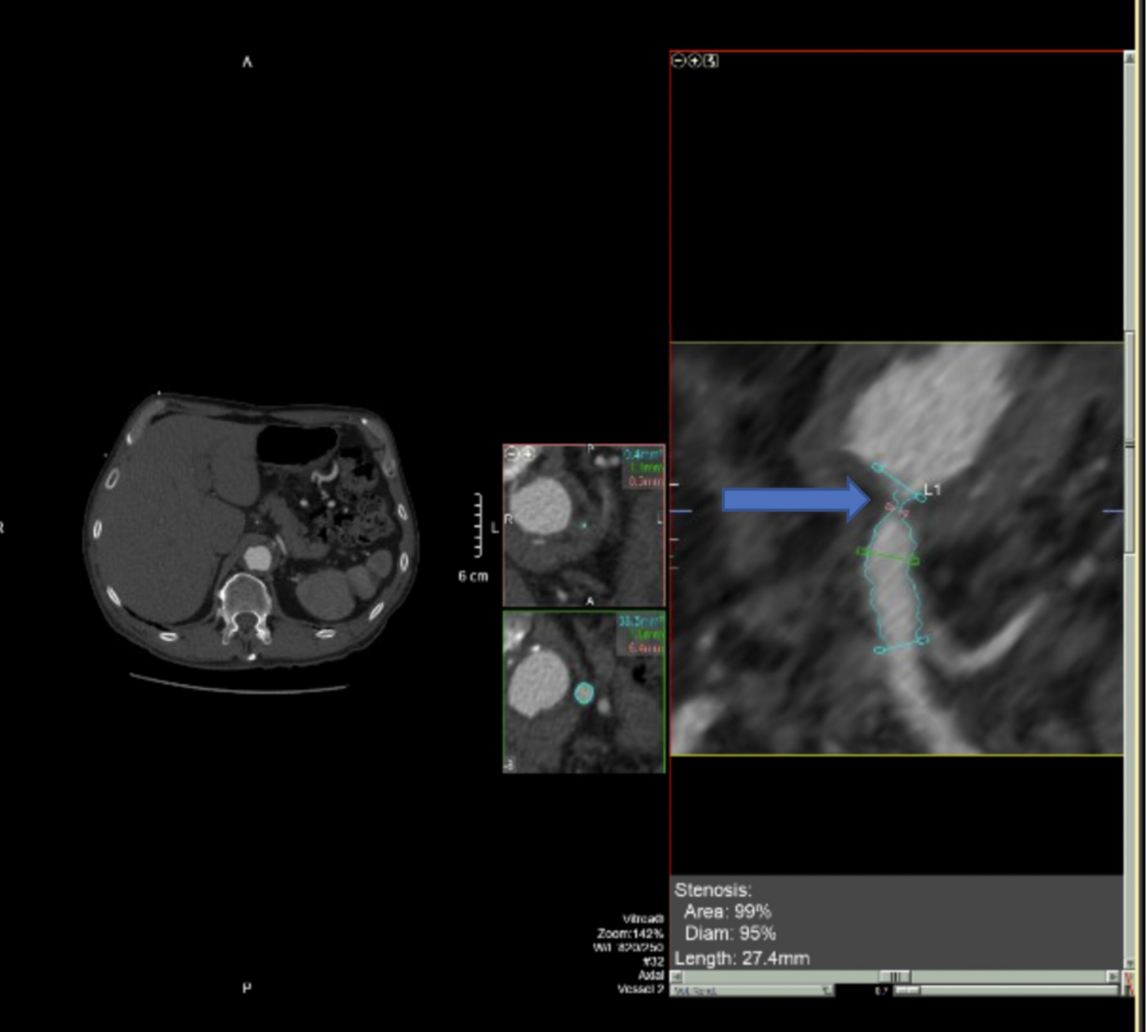
Celiac Artery Stenosis Secondary to Median Arcuate Ligament (arrow)

**Figure 3:**
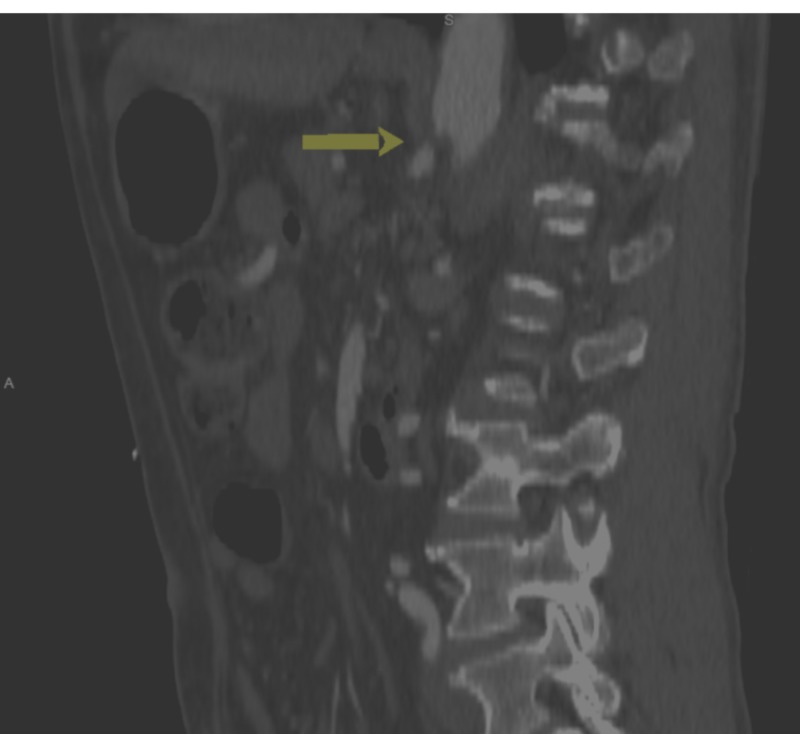
Median Arcuate Ligament Compressing the Celiac Artery

The patient also had newly found celiac artery stenosis in addition to the mesenteric artery occlusion. Interventional radiology (IR) had been consulted for possible stenting of the celiac artery as the patient had intermittent abdominal pain. IR reviewed the CTA images judiciously and concluded that the patient has MALA, and deferred treatment towards surgery service (Figures 1-3). Both general and vascular surgery did not recommend any intervention. The patient's abdominal pain had spontaneously resolved; he had been bridged to warfarin and discharged in a stable state with therapeutic international normalized ratio (INR) of 2-3. The patient did have repeated admissions for the abdominal pain over the next year, and ultimately, he was transferred to a larger tertiary care hospital for intervention; however, he was not offered intervention at that institution as well. During this time, the patient's abdominal pain was intermittent with spontaneous resolution.

## Discussion

The first case of anatomical compression of the celiac axis was reported in 1917 by Lipshutz, followed by a case of MALS in 1963 described by Harjola [3]. Since then, many case studies have been published in this regard. Duran et al. reveal the prevalence of the syndrome to be 2 per 100,000 patients, with a female to male ratio of 2-3:1 [3]. Usually, MAL crosses aorta at L1; above celiac trunk origin [3]. However, in 10% to 24% of the population, deficient caudal migration of the celiac trunk during embryogenesis or abnormally low insertion of MAL results in impingement of the proximal celiac trunk [3].

Origin of celiac trunk occlusion could be classified under intrinsic, congenital, neoplastic, arteriosclerosis, and extrinsic: which is mostly by MAL [9]. This compression was reported to exacerbate when the diaphragm draws down during expiration when the aorta with its branches move over the MAL, further compressing the celiac trunk and therefore resulting in visceral ischemia followed by postprandial abdominal pain [1,9]. Only 1% of patients will experience severe compression, continuing through inspiration [9].

Asymptomatic stenosis has been discovered as accidental findings in up to 6.7% of the general population making clinical presentation a rare instance [2]. Duran et al. argue that the presence of celiac compression in asymptomatic patients implies a secondary factor is involved besides the vascular injury caused compression [3]. The authors also report that at least two or more stenosis are required to lead to ischemia due to the presence of numerous collateral circulations [3]. Impingement by MAL inevitably results in irritation and hyperstimulation of the sympathetic pain fibers of the celiac ganglion, followed by splanchnic vasoconstriction and finally ischemia [1,3]. This is responsible for the chronic pain experienced by patients. Alternate theories describe a steal phenomenon, which is the blood flow from superior mesenteric artery diverting to the celiac artery through collaterals leading to midgut ischemia [1].

Patient experiences pain in the epigastric region, which worsens with the meal, exercise, and leaning forward [1]. Pain may or may not be associated with nausea (9.7%), weight loss (48%), diarrhea (7.8%), and occasionally bloating and emesis, psychologically leading to a fear of food in attempts to avoid symptoms [1,3]. Patients have reported temporary relief of symptoms in the fetal position as this lessens the compression by MAL on the celiac artery [1]. On physical examination, 83% of patients exhibit epigastric bruit, which is aggravated on expiration [1].

Celiac trunk obstruction can result in hemodynamic flow changes proportionate to the degree of occlusion, leading to complications like splanchnic artery aneurysms [8,10]. Any resulting aneurysm requires strict follow up and/or surgical intervention via endovascular approach in conjunction with celiac artery decompression [8]. Prolonged compression results in vasculature changes like intimal hyperplasia, elastic fiber proliferation in media, and disorganization of adventitia [1]. Marked celiac trunk obstruction is predestined to form collaterals to maintain the survival of organs supplied by its branches [10]. Collaterals play a role in preventing foregut ischemic pain, which in turn is responsible for the rarity of MALS clinical presentation [10].

MALS is a diagnosis of exclusion, and hence other visceral pain sources like chronic intestinal ischemia, superior mesenteric artery syndrome, irritable bowel syndrome, biliary causes, and ulcer diseases must be excluded [1,2]. This can be achieved with the help of imaging techniques like mesenteric duplex ultrasonography, CTA, magnetic resonance angiography, gastric tonometry, and mesenteric arteriography can all be used to view the celiac artery compression caused by MAL [1,11,12]. In patients with suspected MALS, a mesenteric ultrasound can be an excellent screening tool showing an elevation in peak systemic velocities during expiration, which normalizes on inspiration or upright posture [1]. On Doppler ultrasound, Aswani et al. set criteria for MALS as a rise in peak systolic velocity (>200 cm/s) with the turbulent flow on expiration [13]. However, a lateral view of angiography remains the gold standard for diagnosis, demonstrating focal constriction of the celiac artery with post stenotic dilations followed by an increment in collaterals from the superior mesenteric artery [1]. This is known as the characteristic hooked appearance visible on CTA and differentiates it from an atherosclerotic lesion due to the absence of calcification and intimal thickening in MALS [1,9,13].

Studies report four surgical interventions opted for the treatment of MALS: celiac artery decompression and celiac ganglionectomy, celiac artery decompression and dilation, celiac artery decompression and reconstruction, and celiac artery endovascular stenting [2]. Decompression and celiac ganglionectomy have long been the widely accepted and conducted operation with the laparoscopic approach rapidly replacing the open decompression [2]. This is primarily due to shorter hospital stay, lesser postoperative complications, less blood loss, reduced time to feeding, better postoperative pain relief, and for cosmetic reasons [2].

Presently, 80% of patients experience immediate relief with surgical decompression via laparoscopic approaches. However, postoperative may mimic MALS symptoms and may require up to six weeks to resolve [1] Moreover, 14% of laparoscopic cases are ineffective due to prolonged and persistent compression, causing remodeling of the arterial wall [4]. These patients, along with those recurrences, benefit with endovascular angioplasty followed by stenting after release [1,4].

## Conclusions

MALS is a multifactorial disease due to chronic compression on vessels and neuronal structures. It presents with vague symptoms of postprandial epigastric pain, nausea, vomiting, and weight loss. It is a diagnosis of exclusion, making workup and interventions challenging. Early diagnosis and treatment can be crucial in maintaining a patient’s quality of life.
